# Polysaccharide extract of *Spirulina* sp. increases effector immune-cell killing activities against cholangiocarcinoma

**DOI:** 10.1371/journal.pone.0312414

**Published:** 2024-10-24

**Authors:** Aussara Panya, Methi Wathikthinnakon, Chutamas Thepmalee, Chutipa Chiawpanit, Suthida Panwong, Yupanun Wutti-in, Preeyanat Vongchan, Phennapha Klangsinsirikul, Pachara Sattayawat, Jeeraporn Pekkoh

**Affiliations:** 1 Cell Engineering for Cancer Therapy Research Group, Faculty of Science, Chiang Mai University, Chiang Mai, Thailand; 2 Department of Biology, Faculty of Science, Chiang Mai University, Chiang Mai, Thailand; 3 Division of Biochemistry, School of Medical Sciences, University of Phayao, Mueang Phayao, Thailand; 4 Doctoral Program in Applied Microbiology, Faculty of Science, Chiang Mai University, Chiang Mai, Thailand; 5 Department of Medical Technology, Faculty of Associated Medical Sciences, Chiang Mai University, Chiang Mai, Thailand; Suez Canal University, EGYPT

## Abstract

Cyanobacteria and algae serving as promising food supplements have recently garnered attention for their emerging potential in anti-cancer activity. Cholangiocarcinoma (CCA) or bile duct cancer is one of the top-leading cancers affecting people, particularly in Asian continent. With patients exhibiting no or minimal symptoms in the early stages, advanced CCA is often diagnosed, and primary treatments such as surgery may not be suitable. Discovery of natural bioactive compounds for cancer treatments have, thus, attracted attention as one of the effective means to combat CCA or to supplement primary treatments. In this work, ethanolic and polysaccharide extracts of cyanobacteria and algae were tested for their cytotoxicity against 2 CCA cell lines (KKU055 and KKU213A). The ethanolic extracts from *Leptolyngbya* sp. and *Chlorella* sp. demonstrated growth inhibition of both CCA cell lines, with IC50 values of 0.658 mg/mL and 0.687 mg/mL for KKU055, and 0.656 mg/mL and 0.450 mg/mL for KKU213A. In contrast, only the polysaccharide extracts from *Sargassum* spp. exhibited a remarkable cytotoxic effect, while the polysaccharide extract from *Spirulina* sp. showed slight effect only at a higher concentration (2 mg/mL). All tested extracts were further investigated for improving immune cell killing ability and showed that *Spirulina* sp. polysaccharide extract was able to improve the immune cell killing ability. This extract was then investigated for its effects on the immune cell population, which demonstrated to have positive impact on NK cell population. To further explore the potential use, synergistic effect of *Spirulina* sp. polysaccharide extract with an already-in-use chemotherapeutic drug, gemcitabine, on immune cell cytotoxicity was investigated. The results showed that the immune cell cytotoxicity was enhanced in the co-treatment compared to the use of each treatment separately. The most apparent difference was observed in KKU055 cells where % living cells were reduced from 78.96% (immune cell alone) to 20.93% when the combined gemcitabine and *Spirulina* sp. extracts were used.

## Introduction

Nature offers unlimited resources of bioactive compounds that benefit us biotechnologically and medicinally and as cancer remains a longstanding global challenge, the reported bioactive activities of cyanobacterial and algal extracts, including their anti-cancer properties, have drawn attention. Mostly, the bioactive compounds from cyanobacteria and algae are polysaccharides, peptides, lipids and pigments [1, 2]. Owing to its commercial availability as a food supplement, *Spirulina* is a well-investigated cyanobacterium for its bioactive compounds including for anti-cancer activity. Having proved its function as an adjunct to chemotherapy to improve immune function and reduce myelosuppression in patients with malignant tumors [3], questions remain whether dietary *Spirulina* has roles in cancer treatments or not. Cholangiocarcinoma (CCA) or bile duct cancer, in particular, has been one of the major cancers that affects people, especially in Asia [4]. Patients typically discover the advancement of early-stage CCA when the cancer has already progressed, as it often manifests without noticeable symptoms initially. To this end, means of cancer treatments, apart from surgery, have been developed to treat CCA including chemotherapy [5].

Looking deep in detail, extracts derived from cyanobacteria and algae could play a major role in anti-cancer activity with their high biodiversity and abundance guaranteeing continuous sources when key bioactive compounds are to be commercialized. Firstly, focusing on common dietary supplements derived from cyanobacteria and algae, such as *Spirulina* and *Chlorella*, ethanolic extracts of *Spirulina platensis* were investigated and shown to inhibit the growth of acute leukemia Kasumi-1 cell lines [6]. Water extracts of *Spirulina platensis* have also shown to inhibit a human lung cancer cell line [7]. Recently, our group has also demonstrated that an ethanolic extract of *Chlorella* sp. promoted cancer cell death, including cholangiocarcinoma cells via inhibition of AKT/mTOR pathway [8]. Though *Sargassum* may not be as well-known as a food supplement compared to the aforementioned *Spirulina* and *Chlorella*, products are commercialized with a recent work reporting cytotoxicity of *Sargassum oligocystom* hydroalcoholic extract on colorectal cancer (CRC) cells [9]. Similarly, *Leptolyngbya* is a cyanobacterium that has only recently drawn attention as a food source with extracts proven to contain anti-proliferative agents against ovarian SK-OV-3 and colon DLD-1 cancer cell lines [10].

Moreover, immunomodulation, aimed at enhancing the ability of immune cells to kill cancers, is a novel strategy that could advance the means of cancer treatment [11]. Adding to this, natural extracts not only exhibit direct anti-cancer activity but have also been shown to enhance immune cell killing activities, leading to improved biological effects in eliminating cancer cells. Evidence supporting the combined use of natural extracts and standard chemotherapeutic drugs has been presented up to the clinical trial level, where natural extracts further enhanced efficiency and reduced side effects when used in combination with chemotherapeutic drugs [12, 13]. To underscore this point even further, it is important to highlight that the combination approach of extracts from cyanobacteria (*Spirulina* sp. and *Leptolyngbya* sp.) and algae (*Chlorella* sp. and *Sargassum* spp.) with standard chemotherapy treatment on modulating immune cell function has not yet been reported.

In this work, we evaluated ethanolic and polysaccharide extracts from cyanobacteria (*Spirulina* sp. and *Leptolyngbya* sp.) and algae (*Chlorella* sp. and *Sargassum* sp.) for their potential anti-cancer activity against CCA cells. Subsequently, the *Spirulina* sp. polysaccharide extract as a potential candidate with the ability to enhance immune cell killing and population was subjected to investigate synergetic effects on immune cell cytotoxicity when combined with gemcitabine. Altogether, it can be concluded that the polysaccharide extract of *Spirulina* sp. increased effector immune cell killing activities against cholangiocarcinoma and the effects were further enhanced when co-treated with a commonly used chemotherapeutic drug, gemcitabine.

## Materials and methods

### Cyanobacteria and algae

*Leptolyngbya* sp. AARL KC45 (hereafter *Leptolyngbya* sp.), *Chlorella* sp. AARL G049 (hereafter *Chlorella* sp.) and *Sargassum* spp. were received from the Algal and Cyanobacterial Research Laboratory, Chiang Mai University, Thailand. The fresh cyanobacterium and algae were dried in an oven and grounded prior to the extraction. *Spirulina (Arthrospira)* sp. (hereafter *Spirulina* sp.) was purchased as a dried powder from Boonsom farm (Mae Wang, Chiang Mai, Thailand).

### Ethanolic and polysaccharide extractions of cyanobacteria and algae

Ethanolic extraction of dried *Leptolyngbya* sp. and *Chlorella* sp. was performed using maceration by which 10 g of dried samples were soaked in 95% (v/v) ethanol and left at room temperature for 24 h. This process was repeated for three times. The solution was then filtered and evaporated. Polysaccharide extraction was performed using hot water extraction (HWE). All dried samples (10 g) were soaked in distilled water at a ratio of 1:25 (w/v) and incubated at 95°C for an hour each, with the process repeated three times. Then, the solution was filtered and evaporated. Ethanol (95% (v/v)) at a ratio of 1:2 (v/v) was added to the crude polysaccharide extracts to precipitate before centrifugation at 6,000 rpm for 5 min. All extracts were kept at -20°C until used. The percent yield was calculated according to the following equation:

$$\text{Percentage}\;\text{of}\;\text{yield}\;=\frac{\text{The}\;\text{weight}\;\text{of}\;\text{the}\;\text{extract}\;\text{(g)}}{\text{The}\;\text{weight}\;\text{of}\;\text{raw}\;\text{material}\;\text{(g)}}\times 100$$

### Polysaccharide extract characterization

To characterize the polysaccharide extract from *Spirulina* sp., the extract was hydrolyzed using 1 N HCl and subjected to High Performance Liquid Chromatography (HPLC) analysis via a service provided by the Sugars and Derivatives Analysis Laboratory, Kasetsart University, Thailand. An HPLC equipped with Agilent Hi-Plex Ca was used to detect glucose, galactose, rhamnose, arabinose, xylose and mannose and quantify in comparison with sugar standards.

### Cell culture

The mCherry (red fluorescent protein) stably expressed CCA cell lines used in this study, including mCherryKKU055 and mCherryKKU213A, were cultured in completed Dulbecco’s Modified Eagle Medium (DMEM)/F-12 Medium (Gibco; Thermo Fisher Scientific, Waltham, MA, USA) (10% fetal bovine serum (FBS) (Gibco; Thermo Fisher Scientific), 1% L-glutamine (Gibco; Thermo Fisher Scientific)) at 37°C in a humidified 5% CO_2_ condition.

Lymphocytes used in this study were obtained from isolated peripheral blood mononuclear cells (PBMCs) of healthy donors. The isolation was performed via gradient density centrifugation using Lymphoprep^TM^ Density Gradient Medium (Gibco; Thermo Fisher Scientific) according to the guidelines of both the Declaration of Helsinki and the University of Phayao Human Ethic Committee, University of Phayao, Phayao, Thailand (approval no. UP-HEC 1.2/010/66). After letting the monocytes adhere to the bottom of culture flask for 4 h, the suspension cells were isolated as lymphocytes and were cultured in AIM-V Medium (Gibco; Thermo Fisher Scientific) at 37°C in a humidified 5% CO_2_ condition overnight prior to the killing assay. All experiments were performed in accordance with relevant guidelines, the Declaration of Helsinki and the University of Phayao Human Ethic Committee, University of Phayao, Phayao, Thailand (approval no. UP-HEC 1.2/010/66).

### Cytotoxicity assay

The cyanobacteria and algae extracts used in this study were the ethanolic extract of *Leptolyngbya* sp., *Chlorella* sp. and *Cordyceps militaris*, and the polysaccharide extract of *Leptolyngbya* sp., *Spirulina* sp., *Chlorella* sp. and *Sargassum* spp. The stocks were prepared at concentrations of 250 mg/mL for ethanolic extracts (dissolved in DMSO) and 50 mg/mL for polysaccharide extracts (dissolved in DI water), which were then diluted in culture media prior to treating the cell cultures at the desired range of concentrations. The cytotoxicity of these extracts against mCherryKKU055 and mCherryKKU213A cells were determined by the crystal violet assay in order to stain the remaining attached cells. Briefly, the mCherry KKU cell lines, were plated 10^4^ cells/well in 96-wells plates and incubated for 24 h before the experiment. Four-fold dilution of all extracts ranging from 2,000 μg/mL to 7.81 μg/mL were used in treatment of each CCA cell line for 24 h and 48 h. After 24 h and 48 h of treatment, the treating supernatant was removed, and the attached cells were stained with crystal violet reagent. After washing and drying, the crystal violet in the attached cells was dissolved in 50% ethanol. The absorbance was read at 595 nm using a microplate reader. The percentages of viable cells calculated from the absorbance value were used in IC50 calculation. The IC50 values were then analyzed in GraphPad Prism 9 software (GraphPad Software, Inc., San Diego, CA, USA).

### Killing assay

A killing assay was performed to screen the extracts that have the potential in increasing the killing activity of lymphocytes. According to cytotoxicity assay result, the concentration of the extracts for killing assay was chosen from the sub-lethal dose (31.25 μg/mL for the ethanolic extracts, 1,000 μg/mL for all polysaccharide extracts, and 7.81 μg/mL for *Cordyceps militaris*). The engineered mCherry expressing KKU055 or KKU213A were plated into 96-well plates (1 × 10^4^ cells/well) 24 h before the experiment. The culture medium was replaced with 50 μL of the 2x of selected concentration of each extract in completed DMEM/F-12. An equal volume of AIM-V media containing lymphocytes was added at effector-to-target (E:T) ratios of 0:1, 10:1 and 20:1. After 48 h of co-culture, the red fluorescent picture from each condition was taken using fluorescence microscope (Eclipse Ts2R-FL, Nikon, Tokyo, Japan), then the remaining attached cells were stained by crystal violet assay. The 595nm-absorbance value was used to calculate cell death percentage.

### Lymphocytes profiling

In order to study the effect on lymphocyte population in co-culture condition, the polysaccharide extract of *Spirulina* sp. and *Chlorella* sp. AARL G049 that demonstrate high killing result were selected for this experiment. The co-culture was performed at E:T ratio 20:1 with 1,000 and 500 μg/mL of the polysaccharide extract of *Spirulina* sp. and *Chlorella* sp. AARL G049. After 48 h of co-culture. The lymphocytes from each condition were collected and equally divided into several microcentrifuge tubes. The lymphocytes in the microcentrifuge tubes were stained with these matched antibodies in order to recognize the cell surface protein marker to study lymphocytes population. The antibodies that were used in this study were anti-human CD3 FITC-conjugated monoclonal antibody (Clone UCHT-1, ImmunoTools, Friesoythe, Germany), anti-human CD4 APC-conjugated monoclonal antibody (Clone MEM-241, ImmunoTools, Friesoythe, Germany), anti-human CD8 APC-conjugated monoclonal antibody (Clone UCHT-4, ImmunoTools, Friesoythe, Germany), anti-human CD16 APC-conjugated monoclonal antibody (Clone LNK16, ImmunoTools, Friesoythe, Germany), IgG1 control APC-conjugated monoclonal antibody (Clone PPV-06, ImmunoTools, Friesoythe, Germany), IgG1 control FITC-conjugated monoclonal antibody (Clone PPV-06, ImmunoTools, Friesoythe, Germany), IgG2a control APC-conjugated monoclonal antibody (Clone PPV-04, ImmunoTools, Friesoythe, Germany). Flow cytometry was performed and analyzed using a CytoFLEX Flow Cytometers (Beckman Coulter, Indianapolis, IN, USA).

### Statistical analysis

All results were collected from 3 independent experiments, and all statistical analyses were performed using GraphPad Prism version 9 software (GraphPad Software, Inc., San Diego, CA, USA). One-way ANOVA analysis and multiple comparison were used in calculation for statistically significant difference between each study group. All bar graphs show the error bars of the standard deviation (SD). A p-value less than 0.05 was considered statistically significant.

## Results

### Cyanobacteria and algae extract induced cholangiocarcinoma cell death

The ethanolic extracts were obtained using maceration with the percent yield of 4.32% and 9.18% for *Leptolyngbya* sp. AARL KC45 and *Chlorella* sp. AARL G049, respectively. Polysaccharide extraction using hot water extraction (HWE) were performed on *Leptolyngbya* sp. AARL KC45, *Spirulina* sp., *Chlorella* sp. AARL G049 and *Sargassum* spp. with the resulted percent yields of 2.32%, 9.69%, 10.24% and 14.12%, respectively.

**Fig 1:**
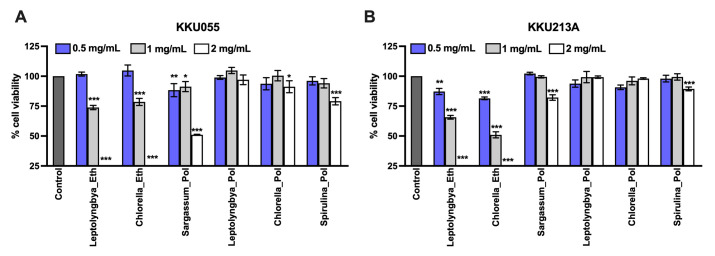
Effects of cyanobacteria and algae extracts on CCA cell viability. The cell viability of KKU055 (A) and KKU213A (B) was measured after the application of the extracts. The ethanolic extracts of *Leptolyngbya* sp., *Chlorella* sp., and polysaccharide extracts of *Sargassum* spp., *Leptolyngbya* sp., *Chlorella* sp., and *Spirulina* sp. at the concentrations of 0.5, 1, and 2 mg/mL were treated in KKU055 and KKU213A. At 48 hours after treatment, cell viability was measured and calculated for % cell viability relative to that of non-treatment control.

The effect of the natural extracts to induce cancer cell death can be applied to the therapeutic approach. Herein, the ethanolic extracts of *Leptolyngbya* sp., *Chlorella* sp., and polysaccharide extracts of *Sargassum* sp., *Leptolyngbya* sp., *Chlorella* sp. *Spirulina* sp. was determined for their cytotoxicity against two CCA cell lines, KKU-213A (well-differentiated) and KKU055 (poorly differentiated). The cells were treated with the extracts at the concentrations of 0.5, 1, and 2 mg/mL for 48 hours. The cell viability was measured and represented as the percentage of cell viability relative to the non-treated control. Comparing ethanolic and polysaccharide extracts, the ethanolic extracts had stronger toxicity to the cells than that of polysaccharide extracts (Fig 1). At 2 mg/mL, the ethanolic extracts derived from *Leptolyngbya* sp. and *Chlorella* sp. eliminated KKU055 and KKU213A completely while the polysaccharide extracts from *Sargassum* sp. and *Spirulina* sp. but not *Leptolyngbya* sp. and *Chlorella* sp. caused the significant cytotoxicity at the equal concentration (Fig 1). Treatment with *Sargassum* sp. and *Spirulina* sp. polysaccharide extracts at 2 mg/mL reduced the cell viability to 51.00% and 82.71% in KKU055 (Fig 1A) where only minor effects were observed in KKU213A with the cell viability of 79.08% and 89.35%, respectively (Fig 1B). The non-linear regression graphs used for IC50 analysis of all extracts against KKU055 and KKU213A cell lines at 48 hours of treatment are also demonstrated in S1 Fig. These results suggested that the ethanolic extracts had more potential anti-cancer activities than the polysaccharide extracts.

### Immunomodulation activity of cyanobacteria and algae extracts to enhance anti-tumor activity of immune cells

**Fig 2:**
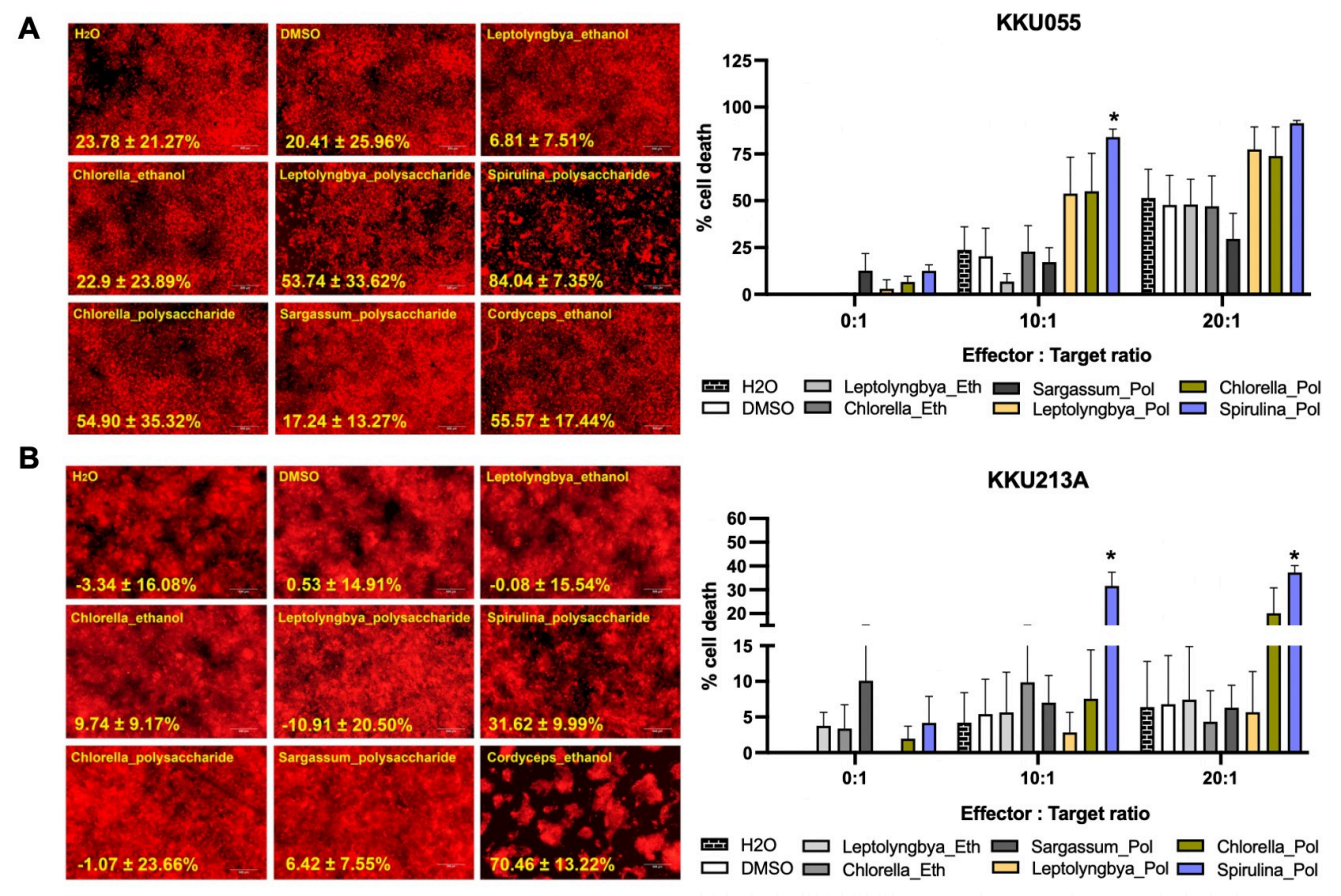
Effects of algae extracts on improving immune cell killing ability. The sublethal-dose ethanolic extracts of *Leptolyngbya* sp., *Chlorella* sp., and polysaccharide extracts of *Sargassum* spp., *Leptolyngbya* sp., *Chlorella* sp., and *Spirulina* sp. were treated in a combination of effector immune cells to KKU055 (A) and KKU213A (B) at the effector to target (E:T) ratio of 0:1, 5:1, and 10:1. The living cancer cells (in red) at 48 hours after co-culturing were determined and used to calculate for % cell death.

We further investigated the immunomodulatory activities of algae extracts to improve the immune cell cytotoxicity against CCA cells. We used the killing assay to determine the killing activity of the immune cells isolated from at least three healthy donors. The engineered red-fluorescence protein expressing CCA cells were treated with the sublethal doses of the ethanolic extracts of *Leptolyngbya* sp., *Chlorella* sp., and polysaccharide extracts of *Sargassum* sp., *Leptolyngbya* sp., *Chlorella* sp. *Spirulina* sp. in combination with immune cells at the effector to target (E:T) ratio of 0:1, 10:1, and 20:1. The percentages of cell death relative to that of non-treated control were judged by the number of living cancer cells after treatment. The results were compared among single treatments (extract or immune cell alone) and combination treatments. Considering the cytotoxicity of immune cells alone, we observed the cytotoxicity of immune cells to eradicate KKU055 and KKU213A in a dose-dependent manner. At the E:T ratio of 10:1 and 20:1, they caused 23.78% and 51.51% in KKU055 (Fig 2A) but were less effective in KKU213A with only 4.1% and 6.39% of cell death (Fig 2B). Interestingly, treatment with polysaccharide extracts but not ethanolic extracts potentially enhanced the immune cell cytotoxicity. Treatment of polysaccharide extracts derived from *Leptolyngbya* sp., *Chlorella* sp. and *Spirulina* sp. with the immune cells augmented the cell death of KKU055 from 23.78% at E:T ratio of 10:1 to 53.74%, 54.90%, and 84.04%, respectively; from 51.51% at E:T ratio of 20:1 to 77.32%, 73.82%, and 91.15%, respectively (Fig 2A). These immunomodulatory effects of *Chlorella* sp. and *Spirulina* sp. were observed in KKU213 evidenced by the increase of KKU213A cell death from 4.1% at E:T ratio of 10:1 to 7.56% and 31.62%; from 6.39% at E:T ratio of 20:1 to 20.12% and 37.29% (Fig 2B).

The polysaccharide extract from *Spirulina* sp. exhibited the most effective effect in promoting immunomodulation effect against CCA cells. Prior to the next experiment, *Spirulina* sp. polysaccharide extract was characterized. The hydrolyzed polysaccharide extract of *Spirulina* sp. was characterized using HPLC and showed to have glucose (61.76%) as a major sugar following by rhamnose (20.59%), arabinose (12.75%) and galactose (4.90%), respectively. The monosaccharide profile obtained is presented in S1 Table.

### Effect of *Spirulina* sp. polysaccharide extract on the immune cell population

**Fig 3:**
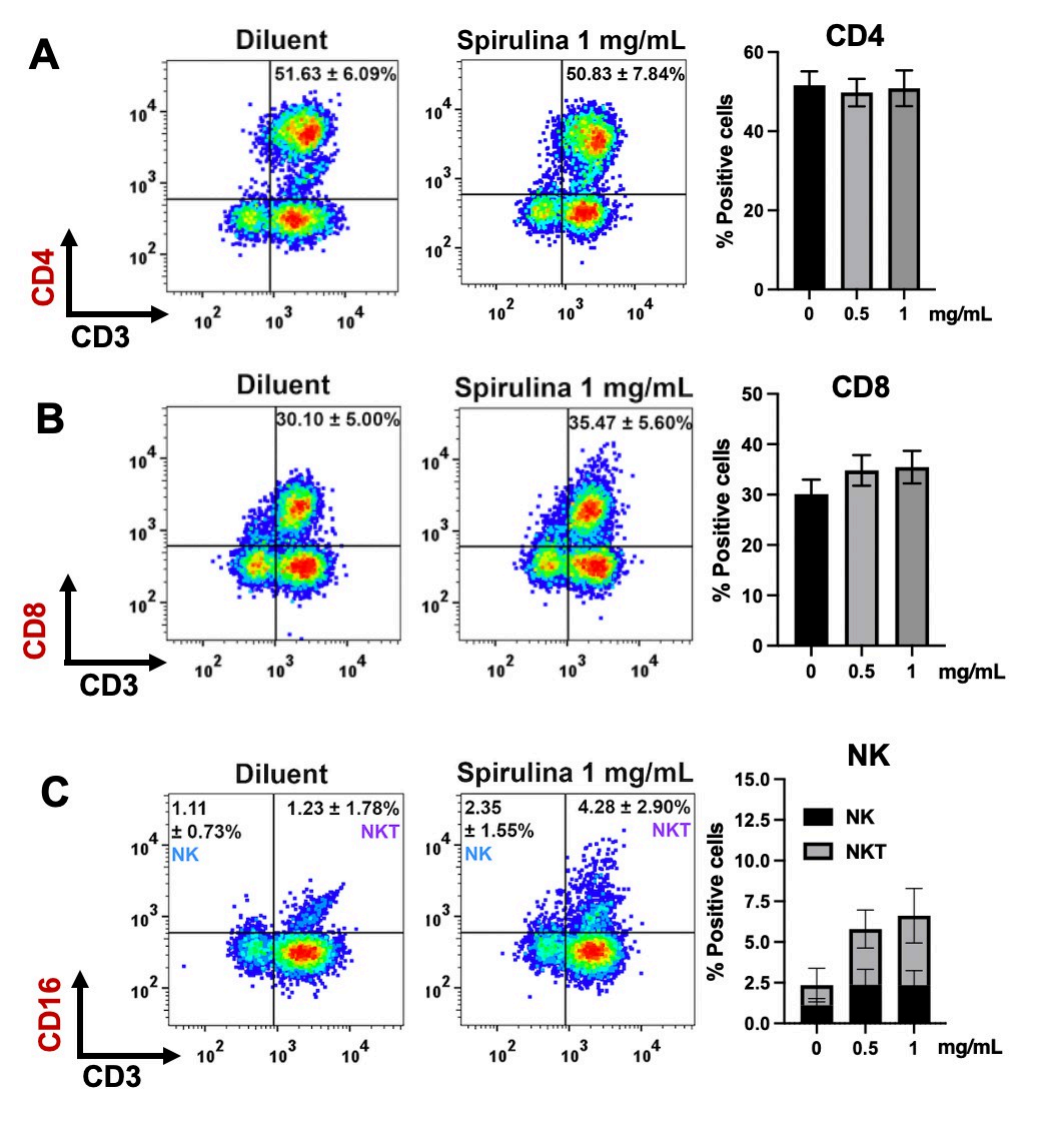
Effect of *Spirulina* sp. polysaccharide extract on the immune cell population. Immune cells were co-cultured with KKU055 at the E:T ratio 20:1 in the presence or absence of 0.5 and 1 mg/mL of the polysaccharide extract of *Spirulina* sp. The cells were harvested after 48 hours of co-culture to determine the proportion of CD4 (CD3^+^CD4^+^) (A), CD8 (CD3^+^CD8^+^) (B), NK (CD3^-^CD16^+^), and NKT (CD3^+^CD16^+^) (C).

We thus further studied the effect of *Spirulina* sp. extract on the immune cell population due to the changes in the immune cell populations or proportion that would explain the mechanism to improve immune cell cytotoxicity, at least in terms of the quality aspect. The population of CD4^+^ T cells, CD8^+^ T cells, NKT cells, and NK cells were determined by using flow cytometry and compared between those before and after polysaccharide treatment (Fig 3). The result showed that 0.5–1 mg/mL of *Spirulina sp*. extract did not significantly affect the proportion of CD4^+^ T cells and CD8^+^ T cells (Fig 3A and 3B). The percentage of CD4^+^ cells was 51.63% whereas treatment with 0.5 and 1 mg/mL extracts yielded 49.76% and 50.83%, respectively (Fig 3A). The CD8^+^ positive cells slightly increased from 30.1% to 34.8% and 35.46% in 0.5 and 1 mg/mL-extract treated cells (Fig 3B). Interestingly, the NK cell population tended to increase after treated with the polysaccharide extract. Treatment with 0.5 and 1 mg/mL extracts increased the numbers of NK positive cells from 3.45% to 8.16% and 8.96% suggesting the possible role of NK cells on anti-tumor activity, particularly after *Spirulina* sp. extract treatment.

### Combination of *Spirulina* sp. polysaccharide extract and gemcitabine additively enhanced immunomodulation effects

**Fig 4:**
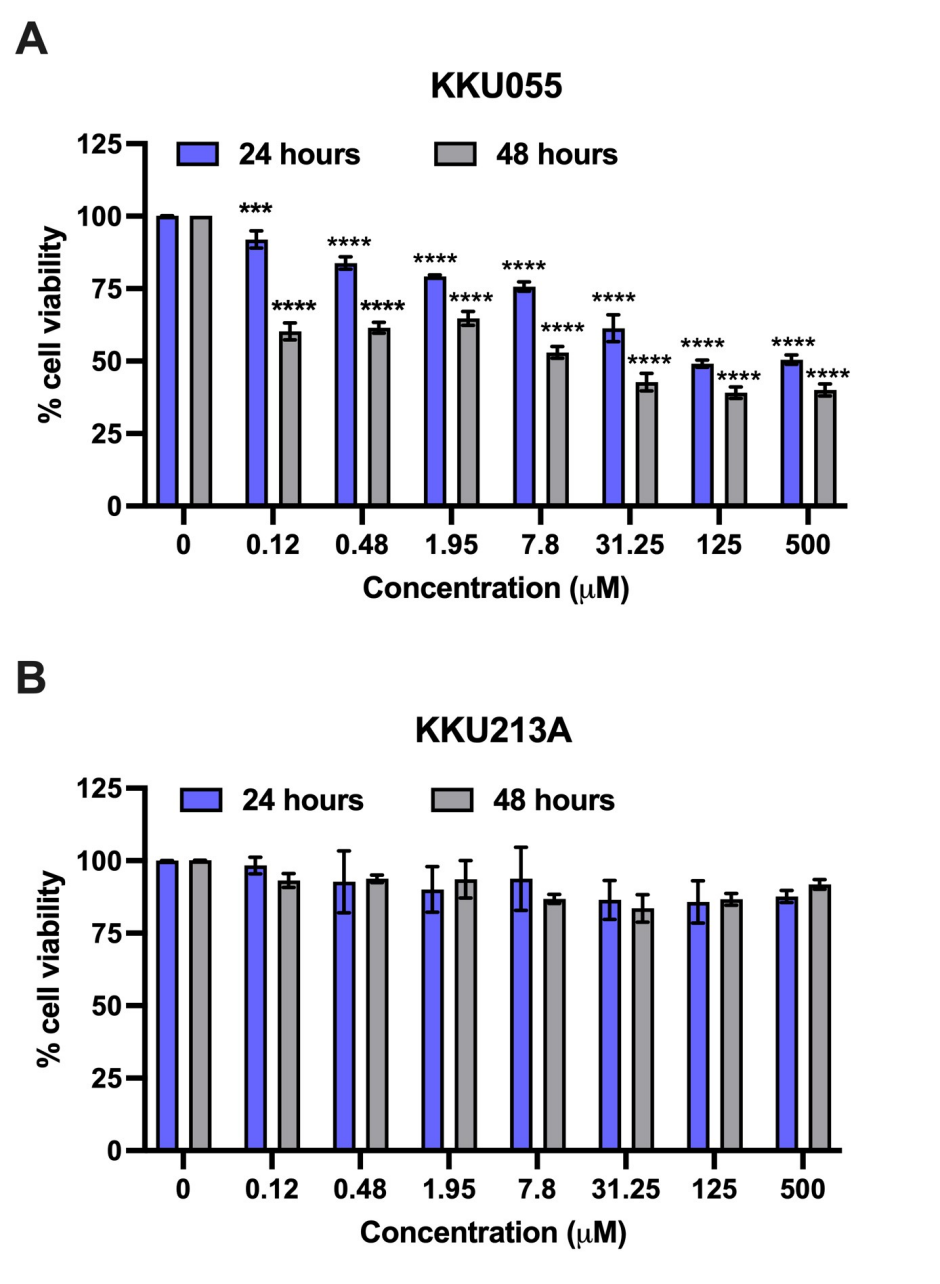
Gemcitabine effects on KKU cell viability. Various concentrations of gemcitabine at 0–500 μM were treated to KKU055 (A) and KKU213A (B). The cell viability was measured after 24 and 48 hours of treatment and used to calculate for % cell viability relative to that of non-treatment control.

Gemcitabine, a standard chemotherapeutic drug, has been reported to enhance immune cell function by upregulating the death receptor on cancer cells. We hypothesized that the combination of gemcitabine to sensitize the cancer cells and polysaccharide extract to modulate the immune cell population could synergistically improve the anti-tumor activity of immune cells. We tested the cytotoxicity of gemcitabine in KKU055 and KKU213A to select the appropriate sublethal doses to test our hypothesis. The cell viability assay revealed the distinct sensitivity of KKU055 and KKU213A to gemcitabine (Fig 4). KKU055 was more sensitive to gemcitabine than KKU213A evidenced by the treatment with gemcitabine at the concentrations ranging from 1.12–500 μM, it caused significant effects only in KKU055 (Fig 4A) but not KKU213A (Fig 4B).

**Fig 5:**
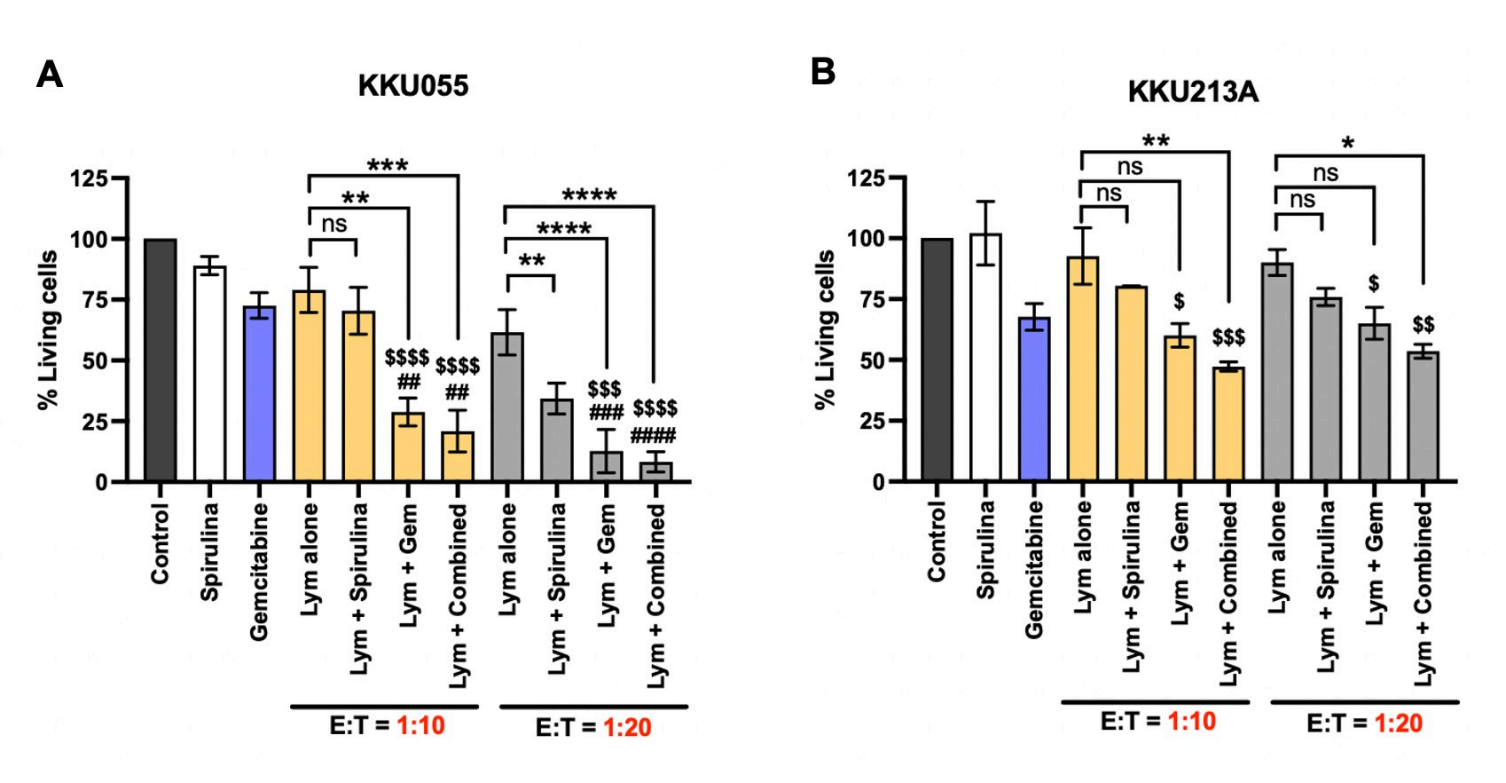
Combination of gemcitabine and *Spirulina* sp. extract potentially improved the immune cell cytotoxicity. The effect of gemcitabine (gem) or *Spirulina* sp. extract alone and combined gemcitabine and *Spirulina* sp. polysaccharide extract (combined) in improving the killing activity of immune cells (lym) were compared in KKU055 (A) and KKU213A (B). The cell viability at the E:T ratio of 10:1 and 20:1 was measured after 24 hours of co-culturing. The numbers of living cells after treatment were used to calculate for % of living cells relative to that of non-treatment control.

We then used a low concentration of gemcitabine (achieved cell viability of more than 70%) to sensitize the cancer cells followed by co-culture with the immune cells at the E:T ratio of 10:1 and 20:1 in the presence or absence of low-dose *Spirulina* sp. polysaccharide extract. The result demonstrated that the combination of gemcitabine and *Spirulina* sp. extract potentially improved the immune cell cytotoxicity against KKU055 and KKU213A (Fig 5). In KKU055 cells, the gemcitabine potentially sensitized the cancer cells to immune cells. At an E:T ratio of 10:1, it reduced the number of living cells to 28.79% compared to that of gemcitabine alone (72.57%) and immune cells alone (78.96%) (Fig 5A). Combining gemcitabine with *Spirulina* sp. extract additionally improved the anti-tumor activity by reducing the living cell proportion to 20.93% (*Spirulina* sp. extract yielded 70.43%). The E:T of 20:1 exhibited a similar trend where the combined gemcitabine with *Spirulina* sp. extract greatly reduced the number of living cells to 8.30% (gemcitabine plus immune cells yielded 12.73%; extract plus immune cells yielded 32.32%) (Fig 5A). In accompanied with KKU055, the result of KKU213A demonstrated that the combined treatment exhibited superior activity (Fig 5B). The gemcitabine and *Spirulina* sp. extract combination treatment with immune cells caused the reduction of living cells to 47.27% which was higher than of gemcitabine plus immune cells (60.06%) and extract plus immune cells (80.41%) (Fig 5B). The enhanced activity was also observed at the E:T ratio of 20:1 but only slightly increased from those obtained from the E:T ratio of 10:1 (Fig 5B). Taken together, the results demonstrated the gemcitabine and *Spirulina* sp. extract combination treatment potentially improved the immune cell cytotoxicity against cancer cells.

### Combination of *Spirulina* sp. polysaccharide extract and gemcitabine increased the expression of death receptor, Fas and TRAIL receptor

**Fig 6:**
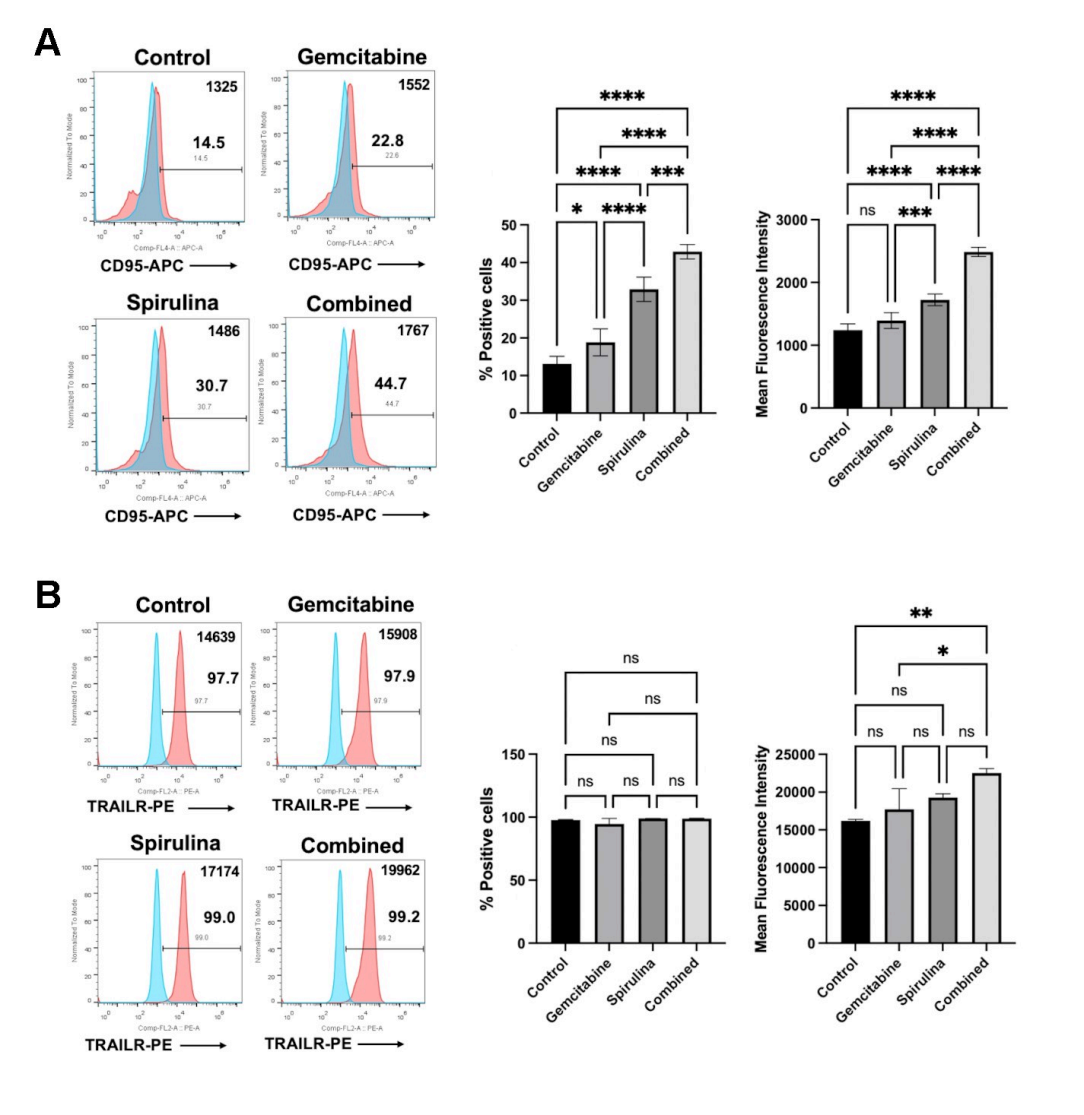
The effect of *Spirulina* sp. polysaccharide extract and gemcitabine or their combination on death receptor expression in KKU055. The effect of gemcitabine (0.5 μM) or *Spirulina* sp. extract (1 mg/mL) alone and combined gemcitabine and *Spirulina* sp. polysaccharide extract (combined) on alteration Fas receptor (CD95) (A) and TRAIL receptor (B) were analyzed by using flow cytometry after 24 hours of treatment.

**Fig 7:**
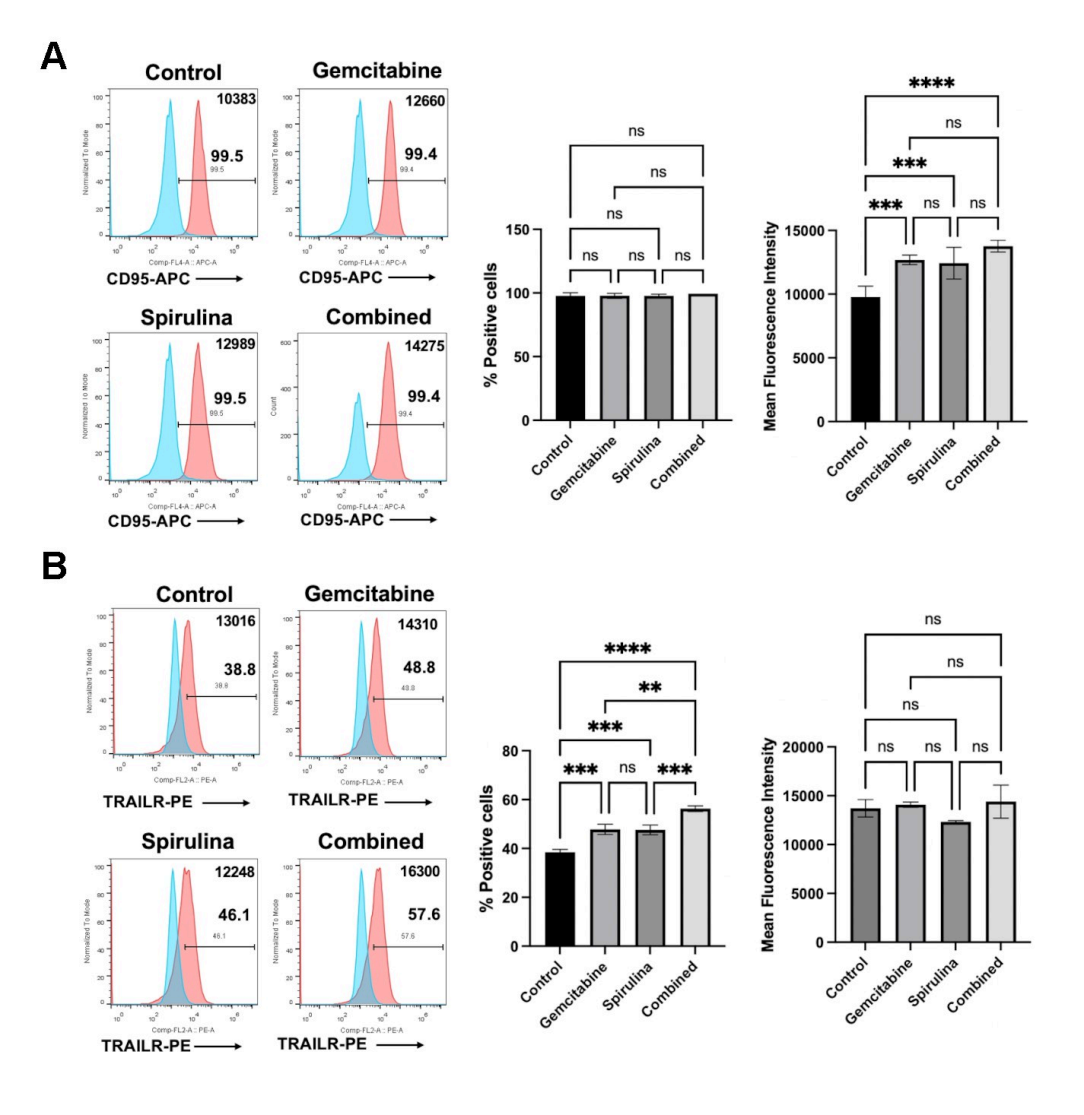
The effect of *Spirulina* sp. polysaccharide extract and gemcitabine or their combination on death receptor expression in KKU213A. The effect of gemcitabine (0.5 μM) or *Spirulina* sp. extract (1 mg/mL) alone and combined gemcitabine and *Spirulina* sp. polysaccharide extract (combined) on alteration Fas receptor (CD95) (A) and TRAIL receptor (B) were analyzed by using flow cytometry after 24 hours of treatment.

Cancer cell sensitization is one mechanism underlying the immunomodulatory activity of chemotherapeutic drugs. Previously, our group reported the effect of gemcitabine on increasing the HLA class I expression and death receptors of the cancer cells [14]. Accordingly, to access the possible mechanism of gemcitabine and its combination with *Spirulina* sp. polysaccharide extract on improving the immune cells cytotoxicity, we determined the expression level of Fas receptor (CD95), TRAIL receptor (DR5), and HLA class I (HLA-ABC) upon 24-hour treatment. The result showed that the combined gemcitabine and *Spirulina* sp. polysaccharide extract significantly increased the expression of Fas receptor and TRAIL receptor in KKU055 (Fig 6) and KKU213A (Fig 7).

In KKU055, the combination treatment increased the proportion of Fas receptor-positive cells from 13.1% to 42.9% which is higher than that of gemcitabine alone (18.8%) and *Spirulina* sp. polysaccharide extract alone (32.9%) (Fig 6A), whereas the TRIAL receptor tended to be increased (Fig 6B). Moreover, the combination slightly increased the Fas receptor expression (Fig 7A) while significantly augmenting the proportion of TRAIL receptor in KKU213A from 38.43% to 56.26% compared to 47.8% with gemcitabine alone and 47.6% with *Spirulina* sp. extract alone (Fig 7B). However, no significant change was observed in the percentage of HLA class-I positive cells upon all tested conditions (S2 Fig).

## Discussion

The use of natural dietary supplementation serves various purposes, leveraging the bioactivities of compounds present in natural sources, including antimicrobial, antioxidant, anti-inflammatory effects, etc. Indeed, anti-cancer bioactivity has attracted profound attention as to develop natural compounds for anti-cancer drugs or supplements to the readily available ones. Generally, cancer treatments involve various approaches, including chemotherapy, which utilizes drugs to inhibit or slow the growth of cancer cells [15]. The use of natural compounds or extracts has, thus, been explored for such purposes. Cyanobacteria- and algae-derived compounds with anti-cancer activity have been reported [16, 17]. Cyanobacteria and algae are photosynthetic organisms that are distributed in several habitats. Discovery of bioactive compounds from these organisms could lead to feasible up-scaled commercialization because of their high biodiversity and high abundance. However, limitations of natural compounds or extracts lie on the practical use as the effectiveness in some cases may not reach the expectation due to their limited solubility and poor absorption [18]. Therefore, in this work, not only we investigated the activity of cyanobacteria and algae extracts, but also the combination uses of the extracts with a commonly use anti-cancer drug, gemcitabine.

Cytotoxicity of ethanolic and polysaccharide extracts of cyanobacteria and algae has been previously investigated against several cancer cell lines. To provide some examples, a recent work on ethanolic extract of *Leptolyngbya* sp. has shown cytotoxicity against several cancer cell lines, A375 (skin), A549 (lung), and Caco-2 (colon), while having small effects on normal Vero cells [19]. Our recent work also showed ethanolic extracts of *Chlorella* sp. to inhibit the growth of 5 top-leading cancer cell lines; lung cancer (A549), cervical cancer (Hela), breast cancer (MCF7), hepatocellular carcinoma (Huh7), and cholangiocarcinoma (CCA; KKU213A) [8]. Reported ethanolic extracts of cyanobacteria and algae have shown carotenoids, phenolics, flavonoids and pigments as potential compounds with anti-cancer activity [19]. To be specific, we recently demonstrated gallic acid and lutein as potential key anti-cancer metabolites from *Chlorella* sp. that induced apoptotic cell death of cholangiocarcinoma via AKT/mTOR signaling pathway [8]. While cyanobacteria- and algae-derived polysaccharides such as fucoidan have been shown to be a key bioactive compound that could trigger cancer cell death in *Sargassum* spp. [20, 21]. Exopolysaccharides of 2 *Chlorella* species; *C*. *zofingiensis* and *C*. *vulgaris*, have been proven to exhibit anti-cancer activity on human colon cancer cell lines HCT8 [22]. It has been only recently that *Leptolyngbya* is a focus of research as it is rich in bioactive phytochemicals. Certainly, relatively small number of studies have been reported in this cyanobacterium, thus it has not been used as a commercialized dietary supplement. Yet the spotlight has been directed to this cyanobacterium as a functional ingredient for various industrial applications in foods, cosmetics, pharmaceuticals, and nutraceuticals [19]. *Spirulina* sp. polysaccharides are well-investigated. They have been shown in several studies (S2 Table). For example, polysaccharides extracted from *Spirulina platensis* also inhibited the growth of gastric cancer cells via modulation of galectin-3 and exhibited cyto/DNA Protection [23]. Interestingly, degree of sulfation of polysaccharides also affected the anti-cancer activity differently by which the highest sulfation resulted in the maximum anti-cancer activity [24]. However, *Spirulina sp*. is renowned for its high protein content, constituting approximately 55–70% of cell dry weight [7]. Consequently, during extraction, some proteins may also be co-extracted, even though hot water extraction (HWE) is the standard method for polysaccharide extraction from *Spirulina* sp. [25]. Certainly, this study employed ethanol precipitation to isolate the polysaccharide fraction from the crude extract. However, it is noteworthy that *Spirulina platensis* protein hydrolysate (at 500 μg/ml concentration) has also demonstrated inhibition of several cancer cells; human liver cancer cells (HepG-2), breast cancer cells (MCF-7), gastric cancer cells (SGC-7901), lung cancer cells (A549), colon cancer cells (HT-29), with >80% inhibition [26]. In the present study, ethanolic extracts of *Leptolyngbya* sp. and *Chlorella* sp. and polysaccharide extracts of *Sargassum* sp., *Leptolyngbya* sp., *Chlorella* sp. and *Spirulina* sp. were investigated (Fig 1). In our investigation, both CCA cell lines—KKU055 and KKU213A—exhibited a consistent trend. It was evident that ethanolic extracts demonstrated higher cytotoxic effects, as all concentrations (0.5, 1, and 2 mg/mL) led to a significant decrease in cell viability compared to the non-treatment control, whereas polysaccharide extracts only showed the similar scenario from *Sargassum* spp. At a higher concentration, 2 mg/mL, the polysaccharide extract from *Spirulina* sp. showed a significant effect on both cell lines. While 2 mg/mL may seem like a high dose, it should be noted that the primary aim is to utilize this extract as a dietary supplement to enhance immune cell function. Its low cytotoxicity indicates high biocompatibility, emphasizing its potential suitability for this purpose. Noticeably, *Sargassum* spp. polysaccharide extract showed to have the most pronounced cytotoxicity among all polysaccharide extracts at 2 mg/mL, this could be because the bioactive polysaccharides that play a crucial role in inhibiting CCA cell viability are fucoidan and alginate, which are only present in brown algae such as *Sargassum* spp. [27]. Indeed, different extraction methods yielding different groups of bioactive compounds, and the inhibition mechanisms of these extracts vary based in the key functional metabolites in each extract.

Immunomodulation activity of these extracts was also investigated in order to assess the ability to enhance anti-cancer activity of immune cells (Fig 2). We demonstrated that polysaccharide extract of *Spirulina* sp. significantly enhanced the immune cells’ killing activity (Fig 2) which might be potentially by altering the quality and quantity of these immune cells. The increase in certain cytokines has been reported to enhance immune function. For example, interleukin-12 (IL-12) and IL-15 produced by activated monocytes can activate NK cells and CD8+ T cells, while interferon-gamma (IFN-γ) promotes the proliferation and activation of NK cells, cytotoxic T cells, and enhances antibody-mediated cellular cytotoxicity. Furthermore, cytokines produced by activated T cells, such as IL-2, are also crucial for the activation and proliferation of NK cells. In our experiment, the polysaccharide extract of *Spirulina* sp. did not significantly affect the CD4+ and CD8+ populations, but it did increase the NK cell population. These results suggest that the pathway-particularly the alteration of cytokine IL-2, IL-12, IL-15, and IFN-γ, which responsible for NK cell proliferation may contribute to the underlying mechanism by which the extract enhances the anti-tumor activity of immune cells. This is in agreement with previous reports on immunomodulation activity of cyanobacteria and algae polysaccharide extracts. To be specific, one study confirmed such activities of polysaccharides of *Chlorella vulgaris* in upregulating IFN-γ and IL-2 in chicken peripheral blood mononuclear cells [28]. Moreover, in the case of *Spirulina* sp. polysaccharide extracts, immunostimulatory effects has been demonstrated in RAW 264.7 macrophage cells and clophosphamide (Cy) treated mice [29]. Interestingly, the result from in vivo experiment demonstrated the effect of *Spirulina* sp. polysaccharide extracts on increasing spleen and thymus index, peripheral white blood cells (PWBC), and peripheral blood lymphocytes (PBL) [29]. In addition, the increase of TNF-α and IFN-γ level in the serum was found in dose dependent manner [29] suggesting the potential effect of polysaccharide extracts on the alteration of cytokine which might contribute to the improving of immune cell proliferation and function.

NK cells, as part of innate immunity, play a role in controlling tumor growth through a balance of activating and inhibitory signals, without requiring prior activation. This allows these immune cells to rapidly and effectively attack cancer cells [30, 31]. The increased number of NK cells upon the extract treatment would result in a higher number of functional NKs leading to the improvement in eradicate the cancer cells. Consistent with our observations, cancer cell death increased as early as 24 hours after co-culture, which aligns with the rapid response time of NK cells rather than T cells, as T cells typically require a longer activation period. However, we cannot exclude the contribution of monocytes and T cells to NK cell proliferation, as they are the primary cytokine-secreting cells The immunomodulatory activity of the polysaccharide ascophyllan, purified from *Ascophyllum nodosum* (*A*. *nodosum*), has been previously reported [32]. Administration of ascophyllan to C57BL/6 mice increased the number of NK cells in the spleen and the number of IFN-γ-producing NK cells. However, when ascophyllan was applied to isolated NK cells in vitro, it increased IFN-γ production but did not promote cell proliferation. This suggests that NK cell proliferation likely requires the assistance of other immune cells. Further investigation is necessary to explore the extract’s effects on each immune cell population and to understand the overall impact on enhancing the anti-tumor activity of immune cells.

Based on our results, the polysaccharide extract had only a slight effect on cancer cell viability, even at high concentrations. Therefore, we hypothesize that its primary function lies in its immune-enhancing properties (at least through promoting NK cell proliferation) rather than cytotoxic effects. This result differs from other natural compounds we have studied, such as cordycepin from *Cordyceps* sp. [33] and genistein from soybeans [34], which exhibited strong cytotoxic effects against CCA. These compounds triggered apoptotic signaling cascades and upregulated death receptor proteins, thereby enhancing NK cell activity and increasing the sensitivity of cancer cells to immune cell attacks. Given these differences in molecular mechanisms, these natural compounds can be combined to increase cancer cell sensitivity and improve immune cell function, potentially providing synergistic effects.

Synergistic effects between natural compounds and therapeutic drugs can be through several mechanisms [35]. In this work, combination of a commonly used anti-cancer drug, gemcitabine, and *Spirulina* sp. polysaccharide extract was investigated whether the combination would enhance the immunomodulation or not. In general, gemcitabine has been proven to be suitable for combination treatment with other chemotherapeutic drugs [36] suggesting its potential enhancement when used with other chemicals. The synergistic effect of gemcitabine and immunotherapy (adoptive effector T cells) has been reported previously from our group [14]. The treatment of gemcitabine in CCA cell lines caused the increase in HLA class I expression and the death receptor, CD95 (Fas receptor) which could help to enhance the antigen presentation of the cancer cells and sensitize the cancer cells to activated T cells, respectively [14]. The results of this study demonstrated that gemcitabine alters the expression of the Fas receptor and TRAIL receptor, which enhances the sensitivity of cancer cells to NK cell-mediated cytotoxicity. This effect could potentially contribute to the synergistic impact of combining gemcitabine with polysaccharide extract treatment. As previously described, the polysaccharide extract alone promotes NK cell proliferation. When combined with gemcitabine, this combination makes the cancer cells more susceptible to NK cell attacks.

Gemcitabine is a commonly used chemotherapeutic drug that is effective towards several types of cancers, including non-small cell lung cancer, pancreatic, bladder, and breast cancer and advanced bile duct cancer [37, 38]. Though considered as a relatively low-toxic and well-tolerated drug, side effects of gemcitabine include myelosuppression [38] and life-threatening complications in rare cases [39]. Moreover, the broad-range response and acquired resistance to gemcitabine contribute to the limitation of drug use in some cancer cell types. With highly heterogeneous characteristics, CCA is one of the cancer cell types that has been reported for its wide-ranging characteristics in chemotherapeutic drug resistance. In our study, KKU055 was more sensitive to gemcitabine, greatly lowering cell viability, while no significant difference was found in KKU213A at the highest tested dose (500 μM). This result is consistent with our previous report on CCA cell lines [40]. Previously, a study by Wattanawongdon et al. in 2015 [41] demonstrated the involvement of multidrug resistance mechanisms in gemcitabine resistance in other CCA cell lines. Gemcitabine-resistant CCA cell lines, developed through stepwise long-term exposure, showed upregulation of proteins related to drug resistance mechanisms, including increased MRP1 expression, activation of the PKC signaling pathway, and NF-κB activation [41]. These changes enhance evasion from apoptosis and increase cancer invasiveness. This data points to the necessity of considering the limitations of single-agent chemotherapeutic treatment for CCA. On the other hands, the use of natural compounds in combination with chemotherapeutic drugs has proven useful resulting in enhanced effects, reduced tumor resistance, and decreased adverse effects [42], particularly as a more well-tolerated treatment for patients. Several reports on co-treatments of therapeutic drugs and naturally derived extracts have been shown. To provide a particular example, noni juice (the fruit of the *Morinda citrifolia* tree) ethanolic extracts were reported to affect cholangiocarcinoma cells in combination with 5-fluorouracil (5-FU) both in in vitro and in vivo studies [43]. Several reports have discussed synergistic effects of gemcitabine with natural extracts including traditional Chinese medicinal herbs [44] and *Pao Pereira* [45], and together with findings from this study, the combination uses of gemcitabine and natural extracts, particularly *Spirulina* sp. polysaccharide extract as a food supplement are further underscored for its practicality.

Considering the safety of natural substances, *Spirulina* sp. extract has been well-documented for its high safety reflecting by its currently used as a food supplement and the evidence from several studies in animal models [46–48]. High feeding levels of *S*. *platensis* (30 g of fresh alga or 10 g of dried alga /kg body weight) in Sprague-Dawley rats diary for 12 weeks caused no adverse effects or toxicity [47]. No signs of effects on behavior (i.e., daily food and water intake), health status (i.e., levels of aspartate aminotransferase, alanine aminotransferase, bilirubin, glucose, creatinine, urea nitrogen, uric acid, albumin, and total protein) or pathological abnormalities in the internal organ of those treated rats were found compared to the control group [47]. In our study, the polysaccharide extract of *Spirulina* sp. yielded 9.69% from dried alga. The highest non-toxic concentration in our study was 5 mg/mL (S3 Fig), which is approximately equivalent to 51.6 g/kg of dried algae–around 5 times higher than the reported dose. Based on this data, the effective dose (less than 2 mg/mL) is safe to use in an *in vivo* model. Given the high safety profile of *Spirulina* sp., its implementation as an extract in clinical applications, particularly in cancer treatment, shows great promise. Given the high safety profile of *Spirulina* sp., its implementation as an extract in clinical applications, particularly in cancer treatment, shows great promise. Interestingly, some studies showed the potential of *S*. *platensis* polysaccharide extract on reversing the adverse effect of chemotherapeutic drugs. Xiao-mei et al. showed that the combination of CTX and *S*. *platensis* polysaccharide extract (100 mg/kg or 200 mg/kg body weight daily) in hepatocellular carcinoma xenograft mice improved the anti-tumor effect compared to that of CTX alone. Moreover, the *S*. *platensis* polysaccharide extract greatly recovered the number of peripheral white blood cells, red blood cells and hemoglobin levels which is the myelosuppression side effect of CTX [48]. Furthermore, pre-treatment of *S*. *platensis* polysaccharide extract (1000 mg/kg body weight daily) before CTX treatment improved the hepatic and renal dysfunction where decreased the histological abnormality of liver and kidney caused by CTX [46]. This data demonstrated that *S*. *platensis* polysaccharide extract in combination with the chemotherapeutic drugs did not disturb the anti-tumor activity but provided the synergistic effect and help to reverse the adverse effect of chemotherapeutic drugs. Concordantly, our study demonstrated the indirect effect of the combined gemcitabine and *S*. *platensis* polysaccharide extract (at a sublethal dose of 1 mg/mL) on enhancing immune cell cytotoxicity, highlighting the potential of *S*. *platensis* polysaccharide extract for clinical use. Notably, before proceeding to clinical trials, it is essential to validate the effectiveness of gemcitabine and *Spirulina* sp. polysaccharide extract in treating CCA, at least in an animal model. To translate this work into applicable uses, it is crucial to consider potential adverse effects of *Spirulina* sp. polysaccharides. Despite its widespread use as a dietary supplement, only 128 reports on allergic reactions were identified from PubMed and Scopus databases [49]. This suggests that allergic reactions to *Spirulina* sp. are relatively infrequent. Furthermore, the interaction between *Spirulina* sp. polysaccharide extract and other medications, particularly gemcitabine in this case, should be thoroughly investigated. However, we did not find such reports. Previously, *Spirulina* as a dietary supplement has been used in cancer patients during chemotherapy and shown to benefit them by decreasing the incidences of myelosuppression [3]. This data revealed the safety of *Spirulina* for human consumption and suggests its potential for clinical trials to evaluate the efficacy of *Spirulina* sp. polysaccharide extract in enhancing immune function to control tumor growth. Like other polysaccharides, the degradation and absorption of this extract pose significant challenges for clinical application due to the biochemical structure of long-chain monosaccharides. Advanced delivery technologies, such as nanoparticle encapsulation, could improve absorption and prolong stability. However, the additional investigations into the quantitative analysis of pharmacokinetics (absorption, distribution, metabolism, and excretion of the drug) and pharmacodynamics (the relationship between extract concentration and its pharmacological effects) are crucial for evaluating the drug’s efficacy in humans.

## Conclusion

Altogether, our findings suggest that the polysaccharide extract from *Spirulina* sp. has potential anti-cancer activity against CCA cells with the ability to increase immune cell killing activity and immune cell population, and the effects were even enhanced when the extract was used in combination with gemcitabine against CCA.

## Supporting information

S1 Table Monosaccharide composition of hydrolyzed polysaccharide extract from *Spirulina* sp. (PDF)

S2 Table A list of reports on the anti-cancer activity of *Spirulina* sp. polysaccharides. (PDF)

S1 Fig The non-linear regression graphs used for IC50 analysis of all extracts against KKU055 and KKU213A cell lines at 48 hours of treatment. (TIF)

S2 Fig The effect of Spirulina sp. polysaccharide extract and gemcitabine or their combination on HLA class-I in KKU055 (A) and KKU213A (B).
(TIF)

S3 Fig Cytotoxicity of *Spirulina* polysaccharide extract on Vero cell at 24 h. (TIF)

## References

[1] Sharma R, Mondal AS, Trivedi N. Anticancer potential of algae-derived metabolites: recent updates and breakthroughs. Futur J Pharm Sci. 2023;9. doi: 10.1186/s43094-023-00492-2

[2] Saadaoui I, Rasheed R, Abdulrahman N, Bounnit T, Cherif M, Al Jabri H, et al. Algae-derived bioactive compounds with anti-lung cancer potential. Mar Drugs. 2020;18. doi: 10.3390/md18040197 32276401 PMC7230368

[3] Ge Y, Kang YK, Dong L, Liu LH, An GY. The efficacy of dietary Spirulina as an adjunct to chemotherapy to improve immune function and reduce myelosuppression in patients with malignant tumors. Transl Cancer Res. 2019;8: 1065–1073. doi: 10.21037/tcr.2019.06.13 35116849 PMC8797399

[4] Qurashi M, Vithayathil M, Khan SA. Epidemiology of cholangiocarcinoma. Eur J Surg Oncol. 2023; 107064. doi: 10.1016/j.ejso.2023.107064 37709624

[5] Wang M, Chen Z, Guo P, Wang Y, Chen G. Therapy for advanced cholangiocarcinoma: Current knowledge and future potential. J Cell Mol Med. 2021;25: 618–628. doi: 10.1111/jcmm.16151 33277810 PMC7812297

[6] Flores Hernandez FY, Khandual S, Ramírez López IG. Cytotoxic effect of Spirulina platensis extracts on human acute leukemia Kasumi-1 and chronic myelogenous leukemia K-562 cell lines. Asian Pac J Trop Biomed. 2017;7: 14–19. doi: 10.1016/j.apjtb.2016.10.011

[7] Czerwonka A, Kaławaj K, Sławińska-Brych A, Lemieszek MK, Bartnik M, Wojtanowski KK, et al. Anticancer effect of the water extract of a commercial Spirulina (Arthrospira platensis) product on the human lung cancer A549 cell line. Biomed Pharmacother. 2018;106: 292–302. doi: 10.1016/j.biopha.2018.06.116 29966973

[8] Sawasdee N, Jantakee K, Wathikthinnakon M, Panwong S, Pekkoh J, Duangjan K, et al. Microalga Chlorella sp. extract induced apoptotic cell death of cholangiocarcinoma via AKT/mTOR signaling pathway. Biomed Pharmacother. 2023;160: 114306. doi: 10.1016/j.biopha.2023.114306 36738497

[9] Al-Aadily IRJ, Bajilan SI, Al-Koofee DAF, Al-Marzoqi AH. Anticancer Effect of Sargassum oligocystom Hydroalcoholic Extract Against SW742, HT-29, WiDr, and CT-26 Colorectal Cancer Cell Lines and Expression of P53 and APC Genes. J Gastrointest Cancer. 2023;54: 62–66. doi: 10.1007/s12029-021-00765-0 35000070

[10] Gara-Ali M, Zili F, Hosni K, Ben Ouada H, Ben-Mahrez K. Lipophilic extracts of the thermophilic cyanobacterium Leptolyngbya sp. and chlorophyte Graesiella sp. and their potential use as food and anticancer agents. Algal Res. 2021;60: 102511. doi: 10.1016/j.algal.2021.102511

[11] Petroni G, Buqué A, Zitvogel L, Kroemer G, Galluzzi L. Immunomodulation by targeted anticancer agents. Cancer Cell. 2021;39: 310–345. doi: 10.1016/j.ccell.2020.11.009 33338426

[12] Okem A, Henstra C, Lambert M, Hayeshi R. A review of the pharmacodynamic effect of chemo-herbal drug combinations therapy for cancer treatment. Med Drug Discov. 2023;17: 100147. doi: 10.1016/j.medidd.2022.100147

[13] Lin SR, Chang CH, Hsu CF, Tsai MJ, Cheng H, Leong MK, et al. Natural compounds as potential adjuvants to cancer therapy: Preclinical evidence. Br J Pharmacol. 2020;177: 1409–1423. doi: 10.1111/bph.14816 31368509 PMC7056458

[14] Sawasdee N, Thepmalee C, Sujjitjoon J, Yongpitakwattana P, Junking M, Poungvarin N, et al. Gemcitabine enhances cytotoxic activity of effector T-lymphocytes against chemo-resistant cholangiocarcinoma cells. Int Immunopharmacol. 2020;78: 106006. doi: 10.1016/j.intimp.2019.106006 31780372

[15] Anand U, Dey A, Chandel AKS, Sanyal R, Mishra A, Pandey DK, et al. Cancer chemotherapy and beyond: Current status, drug candidates, associated risks and progress in targeted therapeutics. Genes Dis. 2023;10: 1367–1401. doi: 10.1016/j.gendis.2022.02.007 37397557 PMC10310991

[16] Abd El-Hack ME, Abdelnour S, Alagawany M, Abdo M, Sakr MA, Khafaga AF, et al. Microalgae in modern cancer therapy: Current knowledge. Biomed Pharmacother. 2019;111: 42–50. doi: 10.1016/j.biopha.2018.12.069 30576933

[17] Shahid A, Khurshid M, Aslam B, Muzammil S, Mehwish HM, Rajoka MSR, et al. Cyanobacteria derived compounds: Emerging drugs for cancer management. J Basic Microbiol. 2022;62: 1125–1142. doi: 10.1002/jobm.202100459 34747529

[18] Garcia-Oliveira P, Otero P, Pereira AG, Chamorro F, Carpena M, Echave J, et al. Status and challenges of plant-anticancer compounds in cancer treatment. Pharmaceuticals. 2021;14: 1–28. doi: 10.3390/ph14020157 33673021 PMC7918405

[19] Phinyo K, Ruangrit K, Pekkoh J, Tragoolpua Y, Kaewkod T, Duangjan K, et al. Naturally Occurring Functional Ingredient from Filamentous Thermophilic Cyanobacterium Leptolyngbya sp. KC45: Phytochemical Characterizations and Their Multiple Bioactivities. Antioxidants. 2022;11. doi: 10.3390/antiox11122437 36552645 PMC9774153

[20] Shiao WC, Kuo CH, Tsai YH, Hsieh SL, Kuan AW, Hong YH, et al. In vitro evaluation of anti-colon cancer potential of crude extracts of fucoidan obtained from sargassum glaucescens pretreated by compressional-puffing. Appl Sci. 2020;10: 1–16. doi: 10.3390/app10093058

[21] Somasundaram SN, Shanmugam S, Subramanian B, Jaganathan R. Cytotoxic effect of fucoidan extracted from Sargassum cinereum on colon cancer cell line HCT-15. Int J Biol Macromol. 2016;91: 1215–1223. doi: 10.1016/j.ijbiomac.2016.06.084 27370748

[22] Zhang J, Liu L, Chen F. Production and characterization of exopolysaccharides from Chlorella zofingiensis and Chlorella vulgaris with anti-colorectal cancer activity. Int J Biol Macromol. 2019;134: 976–983. doi: 10.1016/j.ijbiomac.2019.05.117 31121230

[23] Uppin V, Dharmesh SM, R S. Polysaccharide from Spirulina platensis Evokes Antitumor Activity in Gastric Cancer Cells via Modulation of Galectin-3 and Exhibited Cyto/DNA Protection: Structure–Function Study. J Agric Food Chem. 2022;70: 7058–7069. doi: 10.1021/acs.jafc.2c00176 35670428

[24] Mendhulkar VD, Shetye LA, Khot O. Modulation of the Anti-cancer Activity of Sulfated Polysaccharides, Synthesized in Spirulina platensis, Due to Varying Degree of Sulfation Induced by Nutrient and Physical Stress. J Biol Act Prod from Nat. 2020;10: 275–284. doi: 10.1080/22311866.2020.1806729

[25] Guan F, Fu G, Ma Y, Zhou L, Li G, Sun C, et al. Spirulina polysaccharide-based prebiotic foods preparations-a promising approach for modulating gut microbiota and improving health. J Funct Foods. 2024;116: 106158. doi: 10.1016/j.jff.2024.106158

[26] Wang Z, Zhang X. Characterization and antitumor activity of protein hydrolysates from Arthrospira platensis (Spirulina platensis) using two-step hydrolysis. J Appl Phycol. 2016;28: 3379–3385. doi: 10.1007/s10811-016-0881-9

[27] Zhang R, Zhang X, Tang Y, Mao J. Composition, isolation, purification and biological activities of Sargassum fusiforme polysaccharides: A review. Carbohydr Polym. 2020;228. doi: 10.1016/j.carbpol.2019.115381 31635744

[28] Mirzaie S, Tabarsa M, Safavi M. Effects of extracted polysaccharides from a Chlorella vulgaris biomass on expression of interferon-γ and interleukin-2 in chicken peripheral blood mononuclear cells. J Appl Phycol. 2021;33: 409–418. doi: 10.1007/s10811-020-02301-2

[29] Wu X, Liu Z, Liu Y, Yang Y, Shi F, Cheong KL, et al. Immunostimulatory Effects of Polysaccharides from Spirulina platensis In Vivo and Vitro and Their Activation Mechanism on RAW246.7 Macrophages. Mar Drugs. 2020;18. doi: 10.3390/md18110538 33126624 PMC7692637

[30] Prager I, Watzl C. Mechanisms of natural killer cell-mediated cellular cytotoxicity. J Leukoc Biol. 2019;105: 1319–1329. doi: 10.1002/JLB.MR0718-269R 31107565

[31] Topham NJ, Hewitt EW. Natural killer cell cytotoxicity: How do they pull the trigger? Immunology. 2009;128: 7–15. doi: 10.1111/j.1365-2567.2009.03123.x 19689731 PMC2747134

[32] Zhang W, Okimura T, Oda T, Jin JO. Ascophyllan Induces Activation of Natural Killer Cells in Mice in Vivo and in Vitro. Mar Drugs. 2019;17. doi: 10.3390/md17040197 30925723 PMC6521296

[33] Panwong S, Wathikthinnakon M, Kaewkod T, Sawasdee N, Tragoolpua Y, Yenchitsomanus PT, et al. Cordycepin sensitizes cholangiocarcinoma cells to be killed by natural killer-92 (Nk-92) cells. Molecules. 2021;26. doi: 10.3390/molecules26195973 34641520 PMC8512070

[34] Chiawpanit C, Panwong S, Sawasdee N, Yenchitsomanus PT, Panya A. Genistein Sensitizes Human Cholangiocarcinoma Cell Lines to Be Susceptible to Natural Killer Cells. Biology (Basel). 2022;11. doi: 10.3390/biology11081098 35892954 PMC9330512

[35] Pezzani R, Salehi B, Vitalini S, Iriti M, Zuñiga FA, Sharifi‐Rad J, et al. Synergistic effects of plant derivatives and conventional chemotherapeutic agents: An update on the cancer perspective. Med. 2019;55: 1–16. doi: 10.3390/medicina55040110 30999703 PMC6524059

[36] Lee DE, Kang HW, Kim SY, Kim MJ, Jeong JW, Hong WC, et al. Ivermectin and gemcitabine combination treatment induces apoptosis of pancreatic cancer cells via mitochondrial dysfunction. Front Pharmacol. 2022;13: 1–11. doi: 10.3389/fphar.2022.934746 36091811 PMC9459089

[37] Abdel-Rahman O, Elsayed Z, Elhalawani H. Gemcitabine-based chemotherapy for advanced biliary tract carcinomas. Cochrane Database Syst Rev. 2018;2018. doi: 10.1002/14651858.CD011746.pub2 29624208 PMC6494548

[38] Brockman RW, Anderson EP. Role of gemcitabine in cancer therapy. Metab Inhib. 1963;1: 239–285. doi: 10.1016/b978-0-12-395622-4.50012-4

[39] Hryciuk B, Szymanowski B, Romanowska A, Salt E, Wasąg B, Grala B, et al. Severe acute toxicity following gemcitabine administration: A report of four cases with cytidine deaminase polymorphisms evaluation. Oncol Lett. 2018;15: 1912–1916. doi: 10.3892/ol.2017.7473 29434889 PMC5774463

[40] Wathikthinnakon M, Luangwattananun P, Sawasdee N, Chiawpanit C, Lee VS, Nimmanpipug P, et al. Combination gemcitabine and PD-L1xCD3 bispecific T cell engager (BiTE) enhances T lymphocyte cytotoxicity against cholangiocarcinoma cells. Sci Rep. 2022;12: 1–15. doi: 10.1038/s41598-022-09964-6 35418130 PMC9007942

[41] Wattanawongdon W, Hahnvajanawong C, Namwat N, Kanchanawat S, Boonmars T, Jearanaikoon P, et al. Establishment and characterization of gemcitabine-resistant human cholangiocarcinoma cell lines with multidrug resistance and enhanced invasiveness. Int J Oncol. 2015;47: 398–410. doi: 10.3892/ijo.2015.3019 25998688

[42] Wu J, Li Y, He Q, Yang X. Exploration of the Use of Natural Compounds in Combination with Chemotherapy Drugs for Tumor Treatment. Molecules. 2023;28. doi: 10.3390/molecules28031022 36770689 PMC9920618

[43] Prompipak J, Senawong T, Sripa B, Ketterman AJ, Utaiwat S, Woranam K, et al. Anticancer effects of the combined Thai noni juice ethanolic extracts and 5-fluorouracil against cholangiocarcinoma cells in vitro and in vivo. Sci Rep. 2021;11: 1–15. doi: 10.1038/s41598-021-94049-z 34290264 PMC8295291

[44] Pak PJ, Lee DG, Sung JH, Jung SH, Han TY, Park SH, et al. Synergistic effect of the herbal mixture C5E on gemcitabine treatment in PANC-1 cells. Mol Med Rep. 2021;23: 1–9. doi: 10.3892/mmr.2021.11954 33760105 PMC7974510

[45] Yu J, Drisko J, Chen Q. Inhibition of pancreatic cancer and potentiation of gemcitabine effects by the extract of Pao Pereira. Oncol Rep. 2013;30: 149–156. doi: 10.3892/or.2013.2461 23674070

[46] El-Naggar S, Ibrahim M, El-Tantawi H, Al-Sharkawi I. Pretreatment with the Micro-alga, Spirulina Platensis Ameliorates Cyclophosphamide -Induced Hematological, Liver and Kidney Toxicities in Male Mice. Ain Shams J Forensic Med Clin Toxicol. 2018;30: 1–7. doi: 10.21608/ajfm.2018.18076

[47] Hutadilok-Towatana N, Reanmongkol W, Satitit S, Panichayupakaranant P, Ritthisunthorn P. A subchronic toxicity study of Spirulina platensis. Food Sci Technol Res. 2008;14: 351–358. doi: 10.3136/fstr.14.351

[48] Liu X, Zhang H. Effect of spirulina platensis polysaccharide on hematopoietic recovery and related cytokines in mice with transplanted tumor treated by chemotherapy. Chinese J Integr Tradit West Med. 2002;8: 130–133. doi: 10.1007/bf02934440

[49] Gromek W, Kołdej N, Kurowski M, Majsiak E. Spirulina (Arthrospira platensis): Antiallergic Agent or Hidden Allergen? A Literature Review. Foods. 2024;13. doi: 10.3390/foods13071052 38611357 PMC11012157

