# Supplementary material for: Polysaccharide extract of Spirulina sp. increases effector immune-cell killing activities against cholangiocarcinoma
Source: PLoS One. 2024 Oct 24;19(10):e0312414. doi: 10.1371/journal.pone.0312414 (PMC11500882; doi:10.1371/journal.pone.0312414)
Supplement: S1 Table — (PDF) [file pone.0312414.s001.pdf]

Supplementary Table S1 Monosaccharide composition of hydrolyzed polysaccharide extract from *Spirulina* sp.

Sugar	Amount (mg/mL)	% Composition
Glucose	0.63±0.06	61.76
Galactose	0.05±0.00	4.90
Rhamnose	0.21±0.01	20.59
Arabinose	0.13±0.00	12.75
Xylose	Not detected	Not detected
Mannose	Not detected	Not detected
**Total**	1.02	100
