# Supplementary material for: Polysaccharide extract of Spirulina sp. increases effector immune-cell killing activities against cholangiocarcinoma
Source: PLoS One. 2024 Oct 24;19(10):e0312414. doi: 10.1371/journal.pone.0312414 (PMC11500882; doi:10.1371/journal.pone.0312414)
Supplement: S2 Table — (PDF) [file pone.0312414.s002.pdf]

Supplementary Table S2 A list of reports on the anti-cancer activity of *Spirulina* sp. polysaccharides.

**Extract**	**Strain**	**Cell line**	**Reference**
Polysaccharides	*Spirulina platensis*	Gastric cancer cell	Uppin et al., 2023
Sulfated polysaccharides	*Spirulina platensis*	MCF7 breast cancer cell	Mendhulkar et al., 2020
Polysaccharides	*Spirulina platensis*	Human hepatocellular carcinoma	Al-Badwy et al., 2023
Polysaccharides	*Spirulina* sp.	A375 human melanoma cells	Yang et al., 2012
