# Supplementary figures and images for: Polysaccharide extract of Spirulina sp. increases effector immune-cell killing activities against cholangiocarcinoma
Source: PLoS One. 2024 Oct 24;19(10):e0312414. doi: 10.1371/journal.pone.0312414 (PMC11500882; doi:10.1371/journal.pone.0312414)

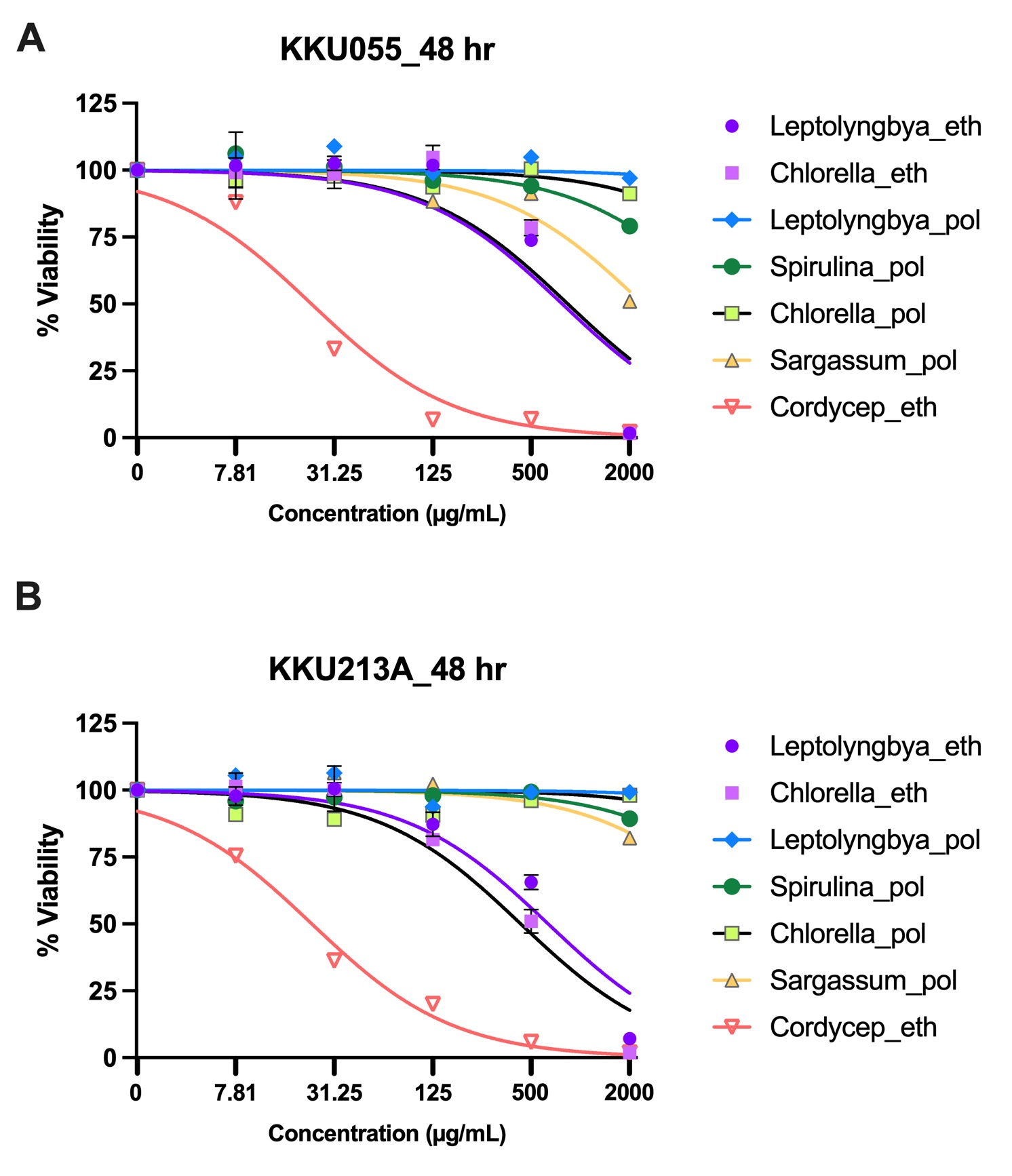

Supplement: S1 Fig — (TIF) [file pone.0312414.s003.tif]

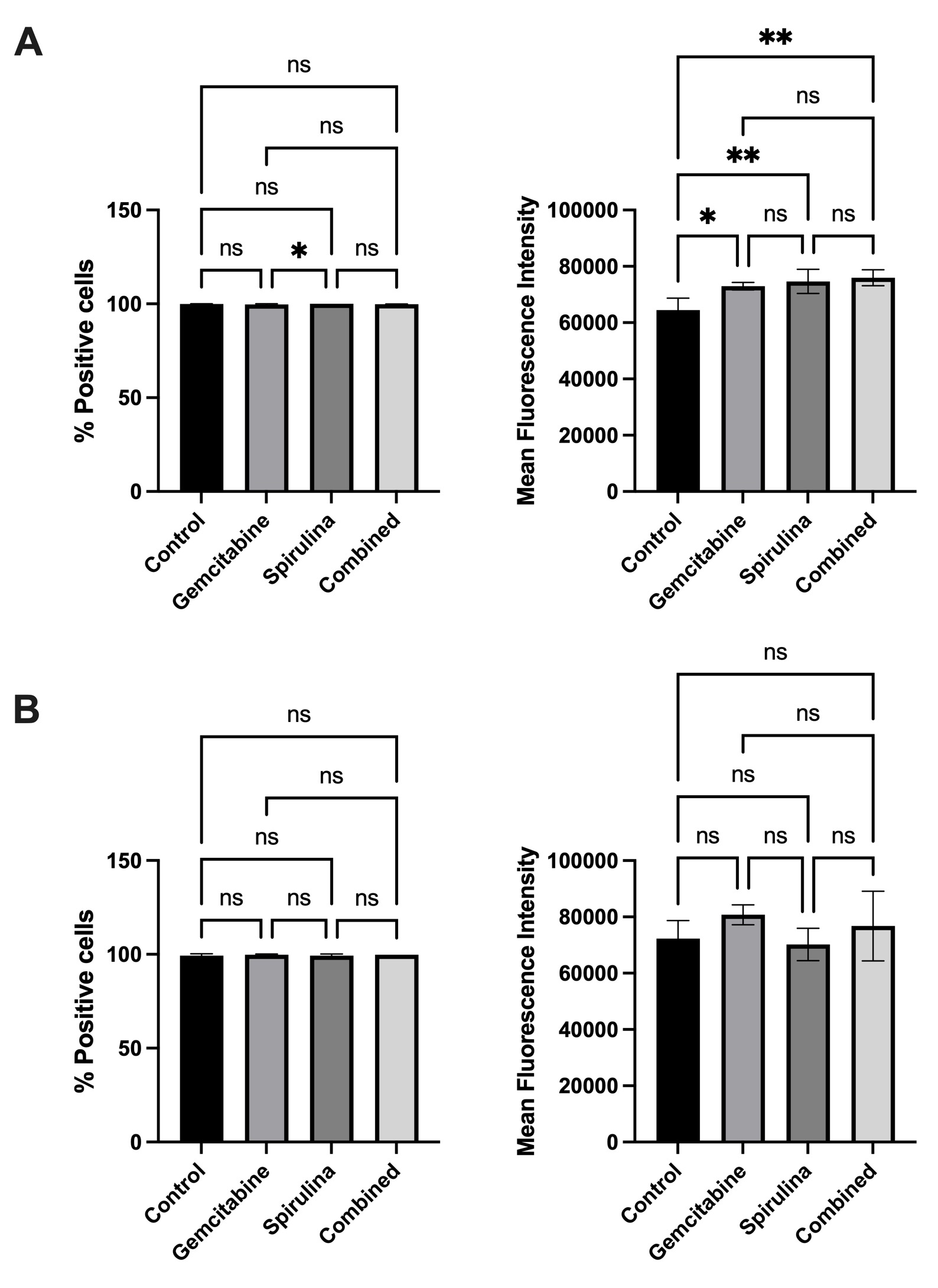

Supplement: S2 Fig — The effect of Spirulina sp. polysaccharide extract and gemcitabine or their combination on HLA class-I in KKU055 (A) and KKU213A (B). (TIF) [file pone.0312414.s004.tif]

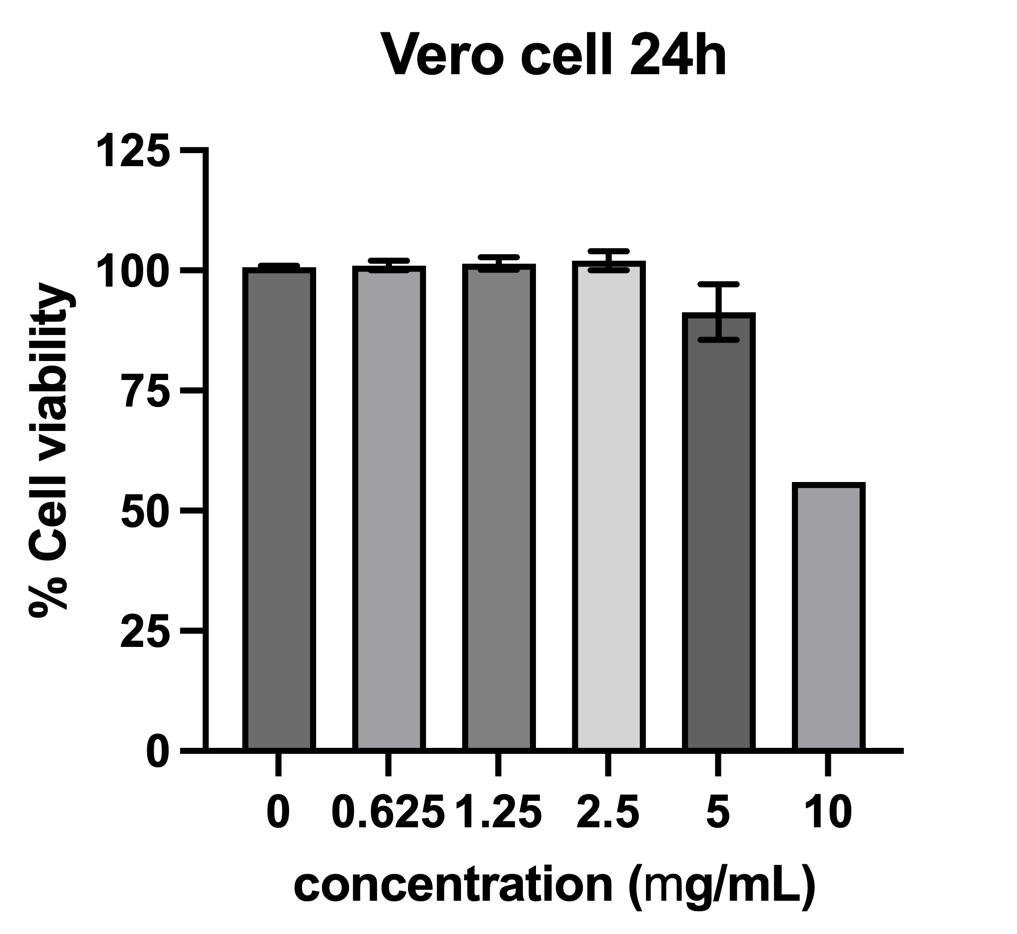

Supplement: S3 Fig — (TIF) [file pone.0312414.s005.tif]
